# Compression of Morbidity 1980–2011: A Focused Review of Paradigms and Progress

**DOI:** 10.4061/2011/261702

**Published:** 2011-08-23

**Authors:** James F. Fries, Bonnie Bruce, Eliza Chakravarty

**Affiliations:** Department of Medicine, Stanford University School of Medicine, 1000 Welch Road, Suite 203, Stanford, CA 94304, USA

## Abstract

The Compression of Morbidity hypothesis—positing that the age of onset of chronic illness may be postponed more than the age at death and squeezing most of the morbidity in life into a shorter period with less lifetime disability—was introduced by our group in 1980. This paper is focused upon the evolution of the concept, the controversies and responses, the supportive multidisciplinary science, and the evolving lines of evidence that establish proof of concept. We summarize data from 20-year prospective longitudinal studies of lifestyle progression of disability, national population studies of trends in disability, and randomized controlled trials of risk factor reduction with life-style-based “healthy aging” interventions. From the perspective of this influential and broadly cited paradigm, we review its current history, the development of a theoretical structure for healthy aging, and the challenges to develop coherent health policies directed at reduction in morbidity.

## 1. Introduction

The Compression of Morbidity paradigm was introduced as a hypothesis of healthy aging in 1980 [1]. It was a counterpoint to the then prevalent paradigm of the “Failures of Success” [2], which argued that increasing life expectancies would lead inevitably to additional years of chronic debilitating illness, economic collapse, and increasing misery for many seniors. In its simplest form, the new thesis was that “the age at first appearance of symptoms of aging and chronic disease can increase more rapidly than life expectancy.” Since most of the morbidity, disability, frailty, infirmity, decreased health-related quality-of-life, medical care costs and other descriptors of ill health (considered here as synonymous) occur later in life and are bounded at the lower end by their age at onset and at the upper end by the age at death; a more rapid rise in the age at first chronic infirmity than in the age at death would squeeze total lifetime morbidity into a shorter span, and thus reduce infirmity [3]. The health strategies necessary to attain morbidity compression, it was conjectured, would be based largely on postponement of ill-health by prevention of chronic disease [4–6]. 

**Figure 1:**
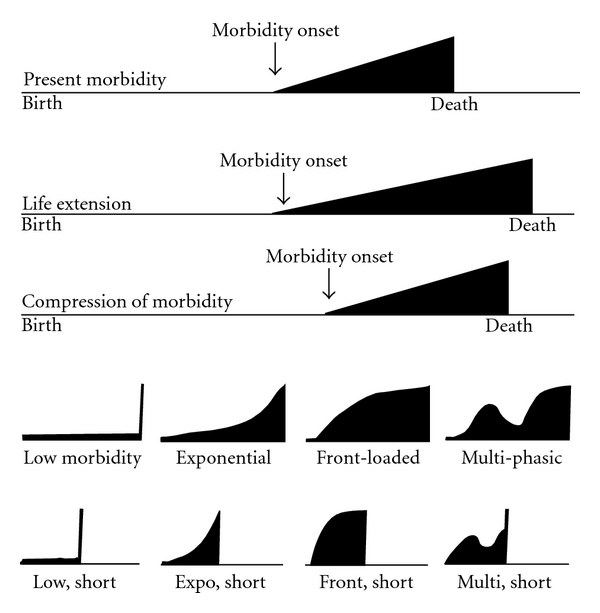
Scenarios for future morbidity. The three major population scenarios in the upper part of the figure represent (1) depiction of a present health, (2) a future where both life expectancy and morbidity are both increased, and (3) a future where both the time period after first morbidity and the amount of morbidity are decreased, resulting in Compression of Morbidity. Shaded areas represent under the curve cumulative morbidity. In the Compression of Morbidity scenario, lifetime disability is decreased. National disability trends over time are the algebraic sum of the eight individual trajectories illustrated at the bottom of the figure and many more. Some individual scenarios may add morbidity to a population average and some may subtract from it. The area under the morbidity curve is a useful metric for population health or health-related quality-of-life. The variables that affect the area under the curve are many and complex and have not yet been well delineated (see text).

Figure 1 extends a common representation of the compression of morbidity [4]. Present lifetime morbidity is contrasted with two of many alternate scenarios, one of life extension and one of morbidity compression. Disability/morbidity in the senior years is displayed as having an initial appearance in later middle age, and to increase linearly over time until death, with lifetime morbidity represented by the area of the triangle. 

Of course, reality is never so uncomplicated. Displayed at the bottom of the figure are a few of the many possible individual life course trajectories which combine to represent population aging [7]. Compression of Morbidity trajectories range from the fatal first heart attack at age 50 (early mortality, minimal morbidity) to the spry 95-year old woman dying asymptomatically in her sleep (late mortality, minimal morbidity). 

An assumption of steady linear decline in function also is too simplistic. There can be recoveries from serious impairments and relapses from successful treatments [8]. Population health over time is a sum of the positive and negative morbidity trajectories of the individuals in the population. We can measure the algebraic sum but not yet the individual trajectories. Trajectories with the greatest cumulative morbidity tend toward early onset intractable morbidity with long periods of limited function, as with the 20-year-old quadriplegic. We remain in the early days of trying to categorize and quantitate the effects of different trajectories of morbidity [7].

A variety of misunderstandings of the compression of morbidity hypothesis characterized the early years of the paradigm. These were influenced by concerns that emphasis on prevention would stifle research in the sciences of aging, that the necessary infrastructure for care of increasing numbers of seniors might not be forthcoming, that demographic projections embarrassingly might require revision, that funding might shift away from technology and specialized care, and that the eventual ultimate achievement of immortality was rendered more suspect [8–15]. Looking back at these criticisms, often offered forcefully, there seems a tendency toward protection of self-interests. A problem with projections of morbidity at that time, of course, was that there were no data at all on trends in disability over time, let alone different trends over time for different population subgroups.

Some specific misunderstandings centered around whether the human life span is fixed and whether compression of morbidity in the future was certain [10]. To set these distractions to rest, compression of morbidity is clearly not inevitable, as has been shown in the Eastern European experience, in many third world countries, and in segments of populations in the developed world. Similarly, average life expectancy from birth, and even from age 65, is expected to continue to increase for many years by almost all observers, including ourselves. Since any limits would be approached asymptotically, any limit would never actually be reached. Further, there will be many more seniors in future developed populations by any computation; these numbers are driven predominately by increasing birth cohorts, in-migration, and increased rates of survival to age 65. Only about 5% of population increases in those over 65 years of age over the next 20 years will be due to 65 year olds living 20 more years rather than 19 [16]. Moreover, increases in life expectancy do not need to slow or to stop for compression of morbidity to occur [17]. Rather, it is the relative rate of increase in morbidity rates and in mortality rates that is the important metric. 

Reduction in lifetime morbidity through postponement involves four strategies, not only the strategy of primary prevention: (1) “Primordial” Prevention prevents the risk factor (not the illness) from developing. For instance, decreasing the number of teenagers who start smoking or preventing childhood obesity represents primordial prevention; (2) Primary Prevention decreases risk factor prevalence, as by stopping smoking, promoting exercise, reducing weight, and reducing hypertension and cholesterol levels; (3) Secondary Prevention is aimed at preventing progression of disease, as in decreasing second heart attacks, congestive heart failure, or complications of diabetes; (4) Tertiary Prevention aims at reduction of morbid states that have already occurred, as with replacement of faulty hips, failed kidneys or livers, or use of a scooter for locomotion. Tertiary Prevention can reduce morbidity but often does not eliminate it. A strategic approach to reduction in lifetime morbidity requires all four approaches and requires that technological inputs from statins for prevention of heart attacks to cataract extraction be included and their positive and negative effects assessed.

Controversy over the Compression of Morbidity hypothesis began to moderate as the large National Long-Term Care Survey and the National Health Interview Survey begun in 1982 largely to answer issues about longitudinal trends in senior health, began to generate data. At the same time, former critics of the Compression Hypothesis began to espouse “Healthy Aging” programs built around exercise and reducing health risks, recognizing that the “Failures of Success concept” [9, 12] had been actually espousing “unhealthy aging,” an awkward position for a gerontologist. Of course apologists for older concepts are still around [18], in part a dispute remains because of confusion about the metrics of morbidity. This paper is termed “focused” since it is concerned with the evolution of a new paradigm and attempts not to directly criticize those who historically impeded this evolution.

## 2. Metrics of Morbidity

 “Mortality” is conceptually clear. However, dictionaries do not agree on the definition of morbidity. At one pole is a premise that morbidity is a broad concept including everything that detracts from health-related quality of life or adds to ill-health or frailty [14]. At the other pole, morbidity is held erroneously to be the number of diagnoses or the number of chronic conditions for an individual. The problems of defining morbidity as a simple count of chronic conditions are easily understood; diagnoses span a broad range of morbidity, from none to incapacitating. Diabetes can be an abnormal blood test or a devastating disease; cancer can be presumably eradicated only to return after years. The diagnostic label has little directly to do with quantitation of morbidity. Moreover, “chronic illnesses” such as osteoporosis, hypertension, or hyperlipidemia are often not morbid conditions. Diagnosis-counting metrics are a metric of convenience, since these data are incidentally captured by many secondary data sources. Secondary data sources, unfortunately, seldom include everything desired for a study. Use of diagnosis-counting metrics has not correlated consistently with morbidity as defined within a disability or frailty framework [19–21]. 

The more useful metric defines the “latent trait” of morbidity as a broad form of disability, frailty, impairment of functioning at activities of daily living (ADL), or other decrease in health-related quality of life. The most common outcome instruments use functional disability as a proxy for morbidity [19, 21, 22]. Even here, most disability scales do not include disability related to vision, hearing, cognition, or psychological distress [23] and hence are conceptually incomplete. The disability proxy, however, can be argued to (1) include much of the morbidity latent trait, (2) be reliably measurable by patient self-report, (3) be a continuous variable permitting sensitive measurement of change and longitudinal study, (4) be consistent with patient concepts of ill-health, and (5) be correlated with global health measures and other measures of physical function [23–28]. Longitudinal study with disability or physical function measures of morbidity in seniors now exceeds 20 years in some instances, as discussed below.

## 3. Mortality

**Figure 2:**
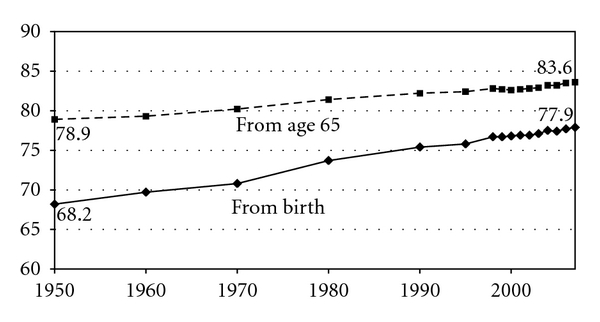
US expected age at death from birth and from age 65, 1950–2007. The lower line shows life expectancy from birth, rising 9.7 years over this period. The upper line takes life expectancy from age 65 and adds 65 years to represent the expected age at death for those who have already survived to age 65; it rises 4.7 years over this period. The lines, while convergent, cannot actually meet unless there are no deaths before age 65. The lines cannot cross; hence the theoretical point of convergent is sometimes termed the “Point of Paradox”.

Mortality changes can be tracked with acceptable accuracy using the Vital Statistics of the United States or other sources [16]. Figure 2 summarizes the US data since 1900, which is generally similar to that of other developed nations. All measures of longevity increase monotonically for almost all years, providing periodic headlines and prophecies of impending crises. The real message, however, is that longevity gains from age 65 and above are quite slow and probably getting slower. 

Curves of expected ages at death calculated from birth and from age 65 (Figure 2) converge slowly and would eventually meet if there were zero deaths under age 65; the curves mathematically cannot cross unless there are fewer than no deaths under age 65, a mathematical impossibility [1, 6, 16]. Estimates of the rate of convergence vary with the base period used for extrapolation; more recent base periods project more distant and higher “Points of Paradox”, where the curves are projected to cross. These curves have been flattening slightly in recent decades, particularly in white females. Women always outlive men, minorities lag; full data are available at the original sources [6, 16]. 

Table 1 shows the average number of years of life remaining from 1900 to 2007 from various ages, combining both sexes and ethnic groups. From birth, life expectancy increased from 49.2 years (previously estimated at 47.3 years in these same sources) in 1900 to 77.9 in 2007, a gain of life expectancy of nearly 29 years and a prodigious accomplishment. The increase was largely due to declines in perinatal mortality and reduction in infectious diseases which affected mainly younger persons. Over this period, developed nations moved from an era of acute infectious disease to one dominated by chronic illness. As a result, life extension from age 65 was increased only 6 years over the entire 20th century; from age 75 gains were only 4.2 years, from age 85 only 2.3 years and from age 100 a single year. From age 65 over the most recent 20 years, the gain has been about a year [16]. 

Much confusion in longevity predictions comes from using projections of life expectancy at birth to estimate future population longevity [18]. For example, “If the pace of increase in life expectancy (from birth) for developed countries over the past two centuries continues through the 21st century, most babies born since 2000 will celebrate their 100th birthdays” [29]. Note from the 100-year line of Table 1 that life expectancies for centenarians would be projected to rise only one year in the 21st century, as in the 20th. Such attention-grabbing statements follow from projecting from birth rather than age 65, thus including infant and early life events to project “senior” aging, using data from women rather than both genders combined, cherry-picking the best data for each year, neglecting to compute effects of in-migration and out-migration, and others. 

Some variant predictions have used “cohort” life tables projected into the future to compare with the “period” life tables that are used traditionally to compare life expectancies. Cohort life tables take a cohort, such as the year 2000, and follow those individuals forward until they have died, perhaps a century from now. Cohort life tables usually result in lower mortality rates. 

Another age exaggeration tactic is to confuse the oldest age obtained with the maximal possible average life expectancy of a population (life span). The first is estimated variously as from 114 to 122 years, the second is perhaps 90 years, at least in this century. Surprisingly, little is learned about a population from its outliers; it is interesting to know the height of the tallest man but it conveys little information about human stature. We believe the population life span (maximal average life expectancy) is the most informative perspective from which to assess human aging. 

The major trespasses are using the 200-year base rather than the last quarter century and the failure to limit study to seniors by basing projections on life expectancy from birth. Of more than peripheral interest here is that proposed revisions of pension systems by increasing the age of eligibility always use the wrong metric, life expectancy from birth. 

Table 2 projects future decedent ages using base rates from 1900 through 2007 (the latest year available at this writing) and a shorter current period, 1980 to 2007, to explore the role of base period in projecting future mortality rates [1, 16]. The computation is straightforward. First, obtain life expectancy values for the base year and last available year, both from birth and from age 65. Second, calculate the average increase in life expectancy per year for each base period and from birth and age 65. Then solve the equations for the Point of Paradox, which identifies the Point at which life expectancy from birth, rising at its rate, is equal to expectancy age from age 65, rising at its slower rate. Over the 107-year base period, the increase in life expectancy from birth over that from age 65 is approximately fourfold; over the 27-year base, it is less than twofold, documenting a flattening of the rate of increase in more recent periods (in the US). The “Point of Paradox” is the point at which the converging lines would cross. With the 107-year base, the Point of Paradox occurs in 2035 at an average age of 85.4. 

This projection, with the long base period, has changed little since we first made this computation in 1980 [1]. On the other hand, with the more recent base period the Paradox occurs in the year 2083 at an average age of 89.8 years. These ages represent an estimation of a maximal attainable average age at death. Given that past trends continue and that no dire disaster nor scientific elixir bends the trend lines. Figure 2 provides some reassurance that this is not an unreasonable assumption, showing stable trends over very long periods. Since the Point of Paradox cannot be reached, the future curve will of necessity move toward an asymptote below the upper trend line and to a point more distant in the future. These and all other projections of future mortality are estimates.

Since 1980 [1], we have performed similar calculations using data from many nations and many baseline periods and from different ages, with congruent results. The maximal average age ranges from 85 to about 93 years, with later base periods tending to be higher, and Japan and several other countries higher than the US We estimated the US maximum average life expectancy at 85 years in 1980, and now at 90 years. US White females currently project to 90.1 years. Thus, given generally stable trends, the maximal attainable mean life expectancy appears to be greater than 90 years and is almost certainly less than 100, far less than the 150 to 200 years still projected by some enthusiasts [29].

These projections hold over a broad range of technical assumptions. The base trend line for these calculations is always life expectancy from birth since this is a coherent place to start and provides the greatest statistical difference in rates. The comparator trend line is most reasonably set at life expectancy from age 65, the traditional age of seniority, for which data are widely available. This age represents for many the boundary between adult life and senior life and is the age at which many senior financial benefits accrue. Since the Point of Paradox occurs at the point where there are no deaths before that age, age 65 works quite reasonably with this assumption since 90 percent of a number of populations already live to age 65. If the comparison age is set lower, such as 55 or 45, the Paradox is reached earlier at a lower age and may actually be below ages already achieved in some populations. If set higher, such as 90 or 100, the Point of Paradox, which cannot be below the top trend line, will be at that number, but not for millennia. Setting the comparison trend from age 75 or 85 gives results similar to 65, a little higher and considerably farther in the future. We believe that the Point of Paradox gives reasonable and transparent estimates for maximal attainable average life expectancy, and that the projections published over 30 years ago remain current today. All estimates by any method, however, are subject to change, and confidence in any estimate should be considered in terms of how many years forward are being extrapolated.

The preceding discussions do not bear directly on the major theme of this paper and discussion of the limits, fixed or not, of life expectancy has been inflammatory and a taboo area for some persons and may have diverted some from considering the central theme of postponement of morbidity. Yet, since the media and the public still are intrigued by the extravagant assumptions, the points made above remain important.

Most importantly, the Compression of Morbidity paradigm does not depend upon whether the human life span is fixed or rising. It depends on relative changes in mortality rates and in morbidity/disability rates [6]. Compression of Morbidity can occur with falling life expectancies, or with rising ones [16].

## 4. Morbidity

Over the past 30 years, three major lines of evidence supporting the Compression of Morbidity Paradigm have emerged, and our research group has been involved in each. First, we began longitudinal studies of disability in seniors in 1984 and 1986 and are now publishing 20-year data on progression of morbidity with age. Second, we encouraged the creation and occasionally advised the National Long-Term Survey and the National Health Interview Survey Projects, examining national trends in the development of senior morbidities. Third, we performed randomized trials of health risk changes after healthy-aging interventions to prove that risk factor reductions and health improvement could be achieved in senior populations. These findings provide proof of concept, that compression of morbidity can occur in specific settings and at specific times. They do not argue that morbidity compression is inevitable. They do provide insight into approaches that might increase our ability to reduce cumulative lifetime morbidity.

## 5. Longitudinal Epidemiologic Studies of Lifestyle and Senior Morbidity

The University of Pennsylvania (UPenn) [30, 31] and the Runners studies [32–34] have reported results periodically and this paper presents an update of progress and findings in these studies. These studies sought to develop data to directly support or refute the Compression of Morbidity hypothesis by prospective longitudinal study of seniors progressing into old age, with development of increasing disability over time. A major prediction of the Compression hypothesis, of course, is that seniors with healthier lifestyles will live longer yet have less cumulative lifetime disability than those with less healthy lifestyles.

Design of such studies contains multiple challenges not the least of which are development of a dedicated and competent staff and collaborators and execution of a plan for continuation of study funding over a very long time; we are grateful for support from three NIH Institutes. The scientific challenges of observational studies are to avoid or control bias and confounding. Our largest concern was about confounding by education, income, and occupation, and associated effects upon smoking, lack of exercise, and obesity, our major lifestyle risks, as well as additional health risks due to poverty, neighborhood, and lack of access to medical care. Accordingly, we recruited populations with favorable social and environmental factors, in part because critics otherwise could suggest that we were just measuring the well-established correlations between poverty and poor health. The UPenn study (1986-) compared University of Pennsylvania alumni from 1939-1940 with 0, 1, or 2 or 3 major health risks. The Runners Study (1984-) compared runners and controls from a university community; both groups were highly educated, lean, and contained few smokers. All data were primary data, collected as an a priori test of the Compression of Morbidity hypothesis.

We followed all subjects yearly with the same protocols, assessing disability with the Health Questionnaire [19, 21], medical history, pain, patient global health, other covariates, and X-rays of knees and hips in subgroups; these were examined for associations with group membership and statistically adjusted when necessary. Women developed more and earlier disability than men, and we repeated analyses for each gender separately. Since some might have taken up running only to discontinue prior to study enrollment, we created secondary study groups of those who had ever run for exercise for more than a month and those who had not and repeated analyses for these “ever-runners” and “never-runners”. Since disability incurred prior to study onset could be important later on, we restricted some analyses to only those cases or controls with zero disability at study onset. All subjects were placed in the study groups at enrollment and remained in the same group subsequently. Study drop-outs were compared with those continuing and found to have similar characteristics.

## 6. University of Pennsylvania Study

**Figure 3:**
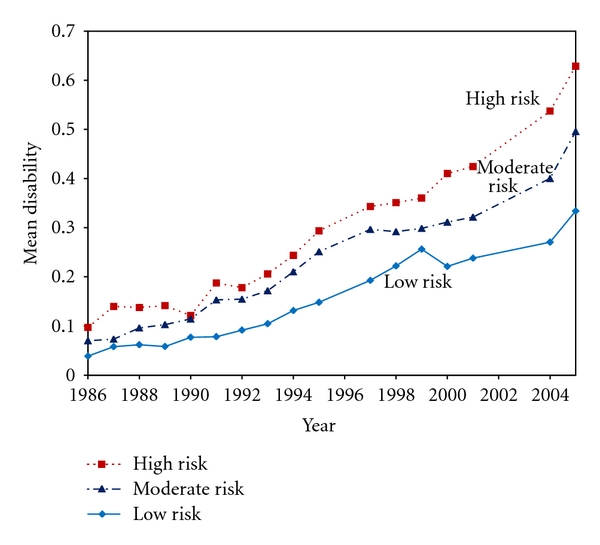
Development of disability with age in seniors after age 68 subjects with low (0), moderate (1) and high (2-3) risk factors in 1986. The University of Pennsylvania Study, over 21 years, has followed seniors from age 68 in three strata: Low Risk, with no baseline risk factors of smoking, obesity, or lack of exercise; Moderate Risk, where one risk factor was present at baseline, and High Risk, where two or three risk factors were present at baseline. The lower line represents low risk subjects, the middle line moderate risk subjects, and the top line high risk subjects. Differences are major whether looked at by vertical differences, or by longitudinal ones (postponement). When contrasted with mortality rates, Compression of Morbidity by health risk factor status is demonstrated across these groups.

**Figure 4:**
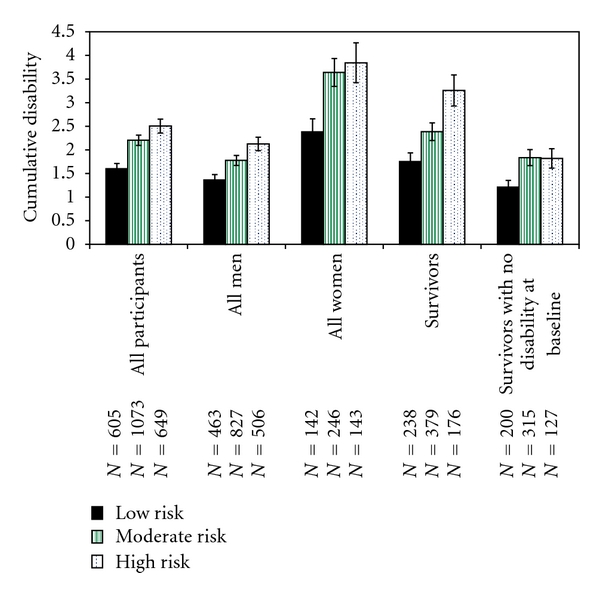
Cumulative disability in seniors by risk groups. Lifetime disability is estimated by summing disability reported from each year for each subject, then averaging by risk group. Results are consistent across men, women, all subjects, survivors, decedents, those with no disability at baseline, and other subgroups at baseline. Greater numbers of risk factors are consistently associated with much more cumulative lifetime disability, a surrogate metric for Compression of Morbidity.

In the University of Pennsylvania study [30, 31], we followed 2,327 alumni from an average age of 68, stratified at baseline into low risk, moderate risk, and high risk groups based upon 0, 1, or 2 or 3 of the three major risk factors (obesity, lack of exercise, smoking). Multivariable survival analyses examined time to disability or death in each group. Postponement of disability and mortality by group were calculated from linear regressions over all data points. Results showed monotonic increases in disability and cumulative disability over age in all groups, with more severe disability (*P* < 0.001) in those with more baseline health risks (Figure 3). Findings were confirmed in men, women, those without initial disability, survivors, and decedents (Figure 4). Disability at the 0.1 level on the Health Assessment Questionnaire was postponed 8.3 years in the low risk groups and moderate disability 5.8 years when compared with the high risk group. Deaths on the other hand were postponed on average only 3.6 to 3.9 years in low risk groups compared with moderate and high risk groups. Results were confirmed by survival analyses adjusted for age, gender, and baseline disability [35]. Good health habits led to greatly increased functional ability, decreased lifetime disability, and longer lives, with effects on morbidity greater than those upon mortality. Compression of Morbidity occurred in the low risk group compared with the high, even in these groups where all subjects were socially advantaged.

## 7. Runners Study

The Runners Study [32–34] is designed to test the effects of regular, vigorous exercise upon morbidity and mortality in seniors. The study was begun in 1984, and 21-year results have been reported; the study was begun because although many studies extol the health benefits of exercise, few if any looked at long-term effects of rigorous exercise in seniors by prospective longitudinal study. The study was designed as a direct test of the Compression of Morbidity hypothesis, positing that exercising individuals, compared with controls, would have prolonged lives but even greater postponement of disability.

We compared 538 members of an over-50 running club with 423 healthy controls also over age 50, beginning in 1984. We obtained radiographs of knees and hips on a 15% subsample of subjects at five-year intervals. Appropriate controls for selection bias were performed including excluding those with baseline disability over zero, completers versus noncompleters, men versus women, and statistical adjustment for major potential confounders. At baseline, Body Mass Index (BMI) differed between groups (23.1 runners, 24.5 controls, both in desirable ranges), runners were younger (58 years versus 63), and runners smoked more rarely (2.1% to 12.9%). Time and cause of death were ascertained from the National Death Index. Details are provided in the original sources [32–34].

**Figure 5:**
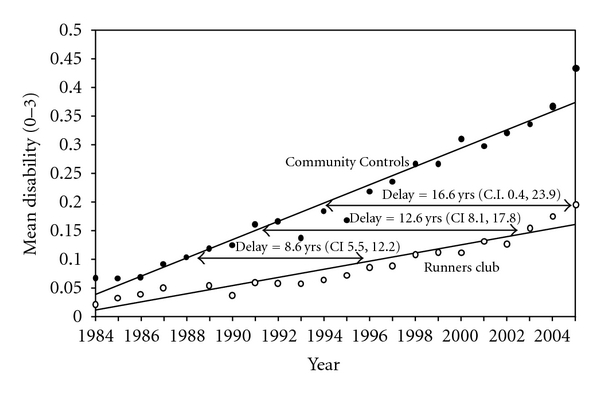
Disability progression—ages 58–79 years: Runners' Club and Community Controls. Progression of disability in Runner's Club and Community Control groups over 21 years from an average age of 58 is compared in the figure both with yearly disability values and statistically derived regression lines. The regression lines are derived from linear mixed models and adjusted for age, gender, BMI, smoking, and initial disability. Comparison of postponement of disability is represented by the absolute difference between the two groups in the time required to develop a given level of disability. The example shown is to reach Health Assessment Questionnaire (HAQ) Disability Index Scores of 0.10, 0.15, and 0.20. All differences are highly statistically different (*P* < 0.001). Lines continue to diverge with age. The postponement is 8.6 years between groups in reaching the  .010 mark, 12.6 years to reach the 0.15 mark, and projected at 16.6 years for the HAQ level of 0.20. Consistent moderately active exercise postpones onset of disability for many years.

Disability was higher in controls throughout the study and differences increased with age (Figure 5). Time to a disability level of 0.15 was postponed 12 years in runners (7 years versus 19 years) representing a postponement of more than a factor of two. Runners trended toward fewer knee and hip replacements than controls (n.s.) [33]. 

Time to death of the first 10% of the sample was postponed in runners by a factor of two (8 years to 16 years) and to a mortality of 15% was postponed from 11.5 years to 19.5 years, less than double. These data are consistent with a marked association of vigorous exercise with both reduced disability and postponed death, with the effect on morbidity marginally greater than the effect on mortality, arguing for modest Compression of Morbidity.

## 8. US National Declines in Disability Levels

The National Long-Term Care Survey (NLTCS) (1982–2004) [36, 37] and National Health Interview Study (NHIS)[20] (1982–1999) are the premier long-term studies of disability trends in the US The NLTCS samples Medicare eligible subjects (*n* = 20,000 per wave) over 65 years of age, whether institutionalized or community-living; the NHIS samples noninstitutionalized individuals over age 70 (*n* = 8,000 per wave). Both show declining disability from 1982 to 2004 with an increase in the rate of decline in more recent years. In the NLTCS, minority declines, which had lagged, also showed declines after 1999 [37, 38]. Of interest, an early critic of the Compression of Morbidity Hypothesis, Kenneth Manton, led the careful analyses and positive interpretations of the NLTCS [37, 38].

Mortality rates over this entire period declined about 1% a year; declines in “any disability” in the NLTCS averaged 1.27% over the entire period and 2.1% in the last five years (Table 3) [37]. Thus, these studies document Compression of Morbidity on a national level, in these populations and over these periods. In all, the seven highest rated US studies over the same periods had similar reductions in disability, although most of these did not cover this entire 22-year period [38–40]. These are the largest, longest, and most carefully performed studies [38–40]. Even so, they are less than desirable. The categories of disability are not clearly defined, the scales are not continuous, and more reliable and sensitive scales are available [26, 28, 41, 42]. Internationally, many (usually smaller and shorter) studies have supported these findings and some not, and it is difficult to identify specific reasons (such as study size, study quality, medical care quality, health risks, or other factors) for differences between studies [40]. Conservatively, we think the positive results in the best studies provide proof of concept, while some discordant results in other populations strongly suggest that improvement in age-specific disability levels over time is not inevitable. No country has yet enunciated or implemented health policies specifically directed at Compression of Morbidity.

## 9. Randomized Controlled Trials in Seniors

The standard for scientific proof of concept is documentation that in randomized controlled trials of theory-based interventions, the intervention provides better outcomes (e.g., greater reduction in disability) than the control intervention.

Relatively few randomized controlled studies of lifestyle change interventions in senior populations have been performed. However, RAND developed an evidence report and evidence-based recommendations for the Center for Medicare and Medical Services, studying 55 programs and rejecting a similar number. Results supported further consideration of Health Risk Appraisal-based programs and recommended the Senior Risk Reduction Program be initiated; it is now ongoing [43]. Other in-depth reviews have yielded similarly positive findings [44–47] and have recommended implementations on a national scale. There is much supporting data [48–50].

Specific studies of successfully well-evaluated programs show common features and results [51–56]. Programs collect Health Risk Appraisal (HRA) data, develop individualized (tailored) reports for each participant, repeat the process at predetermined intervals, incorporate change reports into materials specific for each individual, and use computer techniques to develop individualized interventions. Programs evaluated [57] cost from $6 to $100 per year and have return-on-investment (ROI) from claims savings of 3.5 to 6.1. Health risks have been reduced 10 to 17 percent over the first 6 to 12 months and claims savings per person range up to $570 per year.

The Senior Risk Reduction program, now in its third year, randomly selected 85,000 Medicare recipients aged 67 to 74 and randomly assigned these subjects into two control and two intervention groups. The intervention groups were selected as the most promising interventions from previous studies [43]. Outcomes are being assessed by an independent group and are expected to be available 2011-2012. A positive study will save money for Medicare and improve health outcomes for those in the intervention groups.

## 10. Closing Comments

Compression of Morbidity is a necessary precedent for healthier aging, which in turn completes a vision of improved health throughout the life cycle. Proof of its possibility comes from study of individuals as they course through their senior years, national studies of population aging, and targeted randomized studies of lifestyle interventions in seniors. These specific approaches find support in a rich tapestry of physiologic, psychological, biochemical, medical, and cognitive studies linking lifestyles with health outcomes which flood over scientific publications and other news sources on a daily basis [49, 50].

If we can accomplish morbidity compression without a strategy, as over the past thirty years, then we should be able to further improve if we have a plan. We have identified solid approaches toward such a plan. A strategy should have goals of (1) never smoking, no obesity, never sedentary (Primordial Prevention); the strategy should have goals of (2) increasing exercise, reducing smoking, reducing obesity, and also moderating other health risks (Primary Prevention); (3) within the medical model, goals must include reduction in cholesterol, hypertension, diabetes, time to first heart attack (Secondary Prevention); (4) morbidity-reducing interventions, such as total hip and knee replacements, cataract extractions, and many others (Tertiary Prevention) must also be part of the strategy. Electronic medical records, if (and only if) modified to systematically collect outcome/morbidity/disability data and health risk data, could provide a cost-effective way to develop the data sets necessary to effectively direct policy. These are among the immediate targets and will be enunciated again in the Healthy People 2020 Formulation. There are many more behind them, together of course with the challenges of epidemic illness and man's inhumanity to man. The Compression of Morbidity paradigm is now a comfortable and familiar construct and a base from which to assess health gains and losses.

## Figures and Tables

**Table 1:** US life expectancy 1900–2007 from various ages. Average number of years of life remaining.

From age	1900	1920	1940	1960	1980	2000	2007	Gain, Years 1900–2007
0	49.2	65.4	63.6	69.9	73.9	76.9	77.9	28.7
65	11.9	12.5	12.8	14.4	16.5	17.9	18.6	6.7
75	7.1	7.5	7.6	8.7	10.5	11.3	11.7	4.6
85	4.0	4.2	4.3	4.6	6.0	6.3	n/a	2.3
100	1.6	1.5	2.1	1.9	2.7	2.6	n/a	1.0

**Table 2:** US expected age at death projected from life expectancy data 1900–2007. Projecting future mortality ages: US, all races, both sexes, data 1900–2007 and 1980–2007.

	From birth	From age 65	
Ages at death			
(1900)	49.2	76.9	
(1980)	73.7	81.4	
(2007)	77.9	83.6	
Increase			
(Years: 1900–2007)	28.7	6.7	
(Years: 1980–2007)	4.2	2.2	
Increase/year			
(107 years: 1900–2007)	0.268	0.063	
(27 years: 1980–2007)	0.156	0.081	
Point (Year) of Paradox (where ages of death from birth and from age 65 would be the same) (*X* = Years to Paradox)			
(Projecting from 1900–2007 data—107 years)	85.4	85.4	(2035)
77.9 + 0.268(*X*) = 83.6 + 0.063(*X*), *P* = 2007 + (28 years to Paradox [*P*])			
(Projecting from 1980–2007 data—27 years)	89.8	89.8	(2083)
77.9 + 0.156(*X*) = 83.6 + 0.081(*X*), *P* = 2007 + 76 years			

**Table 3:** US National Long-Term Care Survey (NLTCS): disability categories over time. US population—age 65 and older (%).

Disability category	1982	1989	1994	1999	2004	Overall change	Decline per year (%)
Any	26.5	24.8	23.2	21.2	19.0	−28%	1.27
Mild (IADL only)	5.7	4.5	4.4	3.3	2.4	−58%	2.64
Moderate (1-2)	6.8	6.6	6.1	6.3	5.6	−18%	0.82
Very severe (5-6)	3.5	3.1	2.9	3.0	3.2	−9%	0.41
Institutionalized	7.5	6.9	6.3	4.9	4.0	−47%	2.14
